# Dendritic cells and regulatory T cells expressing CCR4 provide resistance to coxsackievirus B5-induced pancreatitis

**DOI:** 10.1038/s41598-019-51311-9

**Published:** 2019-10-14

**Authors:** Marcela C. S. Françozo, Frederico R. C. Costa, Isabel C. Guerra-Gomes, João S. Silva, Renata Sesti-Costa

**Affiliations:** 1 Institute of Pathology, Hannover Medical School, Hannover, Germany; 2 Department of Biochemistry and Immunology, Ribeirão Preto Medical School, University of São Paulo - USP, Ribeirão Preto, São Paulo, Brazil; 3 Fiocruz- Bi-Institutional Translational Medicine Project, Ribeirão Preto São Paulo, Brazil; 4 Hematology Center, University of Campinas – UNICAMP, Campinas, São Paulo, Brazil

**Keywords:** Antimicrobial responses, Chemokines, Conventional dendritic cells, Viral infection

## Abstract

Type B coxsackieviruses (CVB) are enteroviruses responsible for a common infectious myocarditis and pancreatitis. DCs and regulatory T cells (Tregs) are key players in controlling virus replication and regulating the immune response and tissue damage, respectively. However, the mechanisms underlying cellular migration to target tissues remain unclear. In the present study, we found that CVB5 infection induced CCL17 production and controlled the migration of CCR4^+^ DCs and CCR4^+^ Tregs to the pancreatic lymph nodes (pLN). CVB5 infection of CCR4^−/−^ mice reduced the migration of the CD8α^+^ DC subset and reduced DC activation and production of IFN-β and IL-12. Consequently, CCR4^−/−^ mice presented decreased IFN-γ-producing CD4^+^ and CD8^+^ T cells, an increased viral load and more severe pancreatitis. In addition, CCR4^−/−^ mice had impaired Treg accumulation in pLN as well as increased T lymphocyte activation. Adoptive transfer of CCR4^+^ Tregs but not CCR4^−^ Tregs was able to regulate T lymphocyte activation upon CVB5 infection. The present data reveal a previously unknown role for CCR4 in coordinating immune cell migration to CVB-infected tissues and in controlling subsequent pancreatitis. These new insights may contribute to the design of future therapies for acute and chronic infection of non-polio enteroviruses.

## Introduction

Coxsackieviruses (CVB) belong to the *Picornaviridae* family of the enterovirus genus and circulate worldwide. Their positive sense single-stranded RNA acts as a mRNA after cell entrance, which allows for a fast replication circle. The existing serotypes of CVB are divided into two groups, A and B, according to their pathogenicity. CVB B are cytolytic viruses that represent one of the most common causes of acute infectious myocarditis^1,2^. CVB also have a tropism to pancreatic acinar cells, causing pancreatitis characterized by intense inflammatory infiltration, edema and necrosis of the exocrine pancreas^3^. In addition, some serotypes of CVB are associated with accelerated development of type I diabetes^4,5^.

The innate immune response to CVB is characterized by the recognition of viral proteins and RNA by dendritic cells (DCs), leading to the secretion of type I interferon (IFN-I); this induces an antiviral state, as mice lacking the IFN-I receptor succumb to infection with a very low inoculum of CVB^6,7^. Additionally, it has been demonstrated that bone marrow-derived DCs migrate to the myocardium upon CVB3 infection^8^, and their activation results in IFN-γ and IL-2 production by T cells^9^. Although Th1-mediated responses contribute to tissue damage, IFN-γ-producing cells are required to control CVB replication and therefore resolve the infection^10–12^, whereas IL-17-producing T cells exacerbate disease^13^. Thus, the priming and polarization of T cells, as well as their regulation, are key factors influencing the outcome of CVB-induced disease. Moreover, we previously showed the crucial role of regulatory T cells (Tregs) in controlling exacerbated tissue inflammation induced upon CVB5 infection, which is deleterious to the host. In fact, we and others have shown the importance of adequate T cell polarization in controlling viral spread and tissue injury upon CVB infection^14–16^.

The outcome of infection depends on the migration of immune cells to the target tissues, which is mainly coordinated by chemokines; However, the role of chemokines in cell migration upon CVB infection remains elusive. CCR4 is a chemokine receptor that binds to CCL17 and CCL22 and is expressed by diverse cell types that drive the immune response to CVB, such as DCs and Tregs^17–20^, which prompted us to ask whether CCR4 and its ligands are involved in CVB-induced disease. The present study shows that CCL17 is secreted by pancreatic cells upon CVB5 infection, which is responsible for the migration of CCR4^+^ cells to the pancreatic lymph nodes (PLN). CCR4^+^ DCs and CCR4^+^ Tregs cooperate to promote resistance to CVB5-induced pancreatitis. CCR4^+^ DCs act by inducing a Th1 immune response and IFN-β production, which are important for viral immunity and for the control of pancreatic damage. CCR4^+^ Tregs, in turn, are responsible for regulating T lymphocyte activation, which is known to be crucial for impairing immune-mediated pancreatic injury. Our data show a previously unknown role for a chemokine receptor in orchestrating key cell migration during coxsackievirus infection, which is essential for the cells to exert their function and to influence the outcome of disease.

## Results

### CVB5 infection induces CCL17 production and CCR4^+^ DC migration and activation

**Figure 1:**
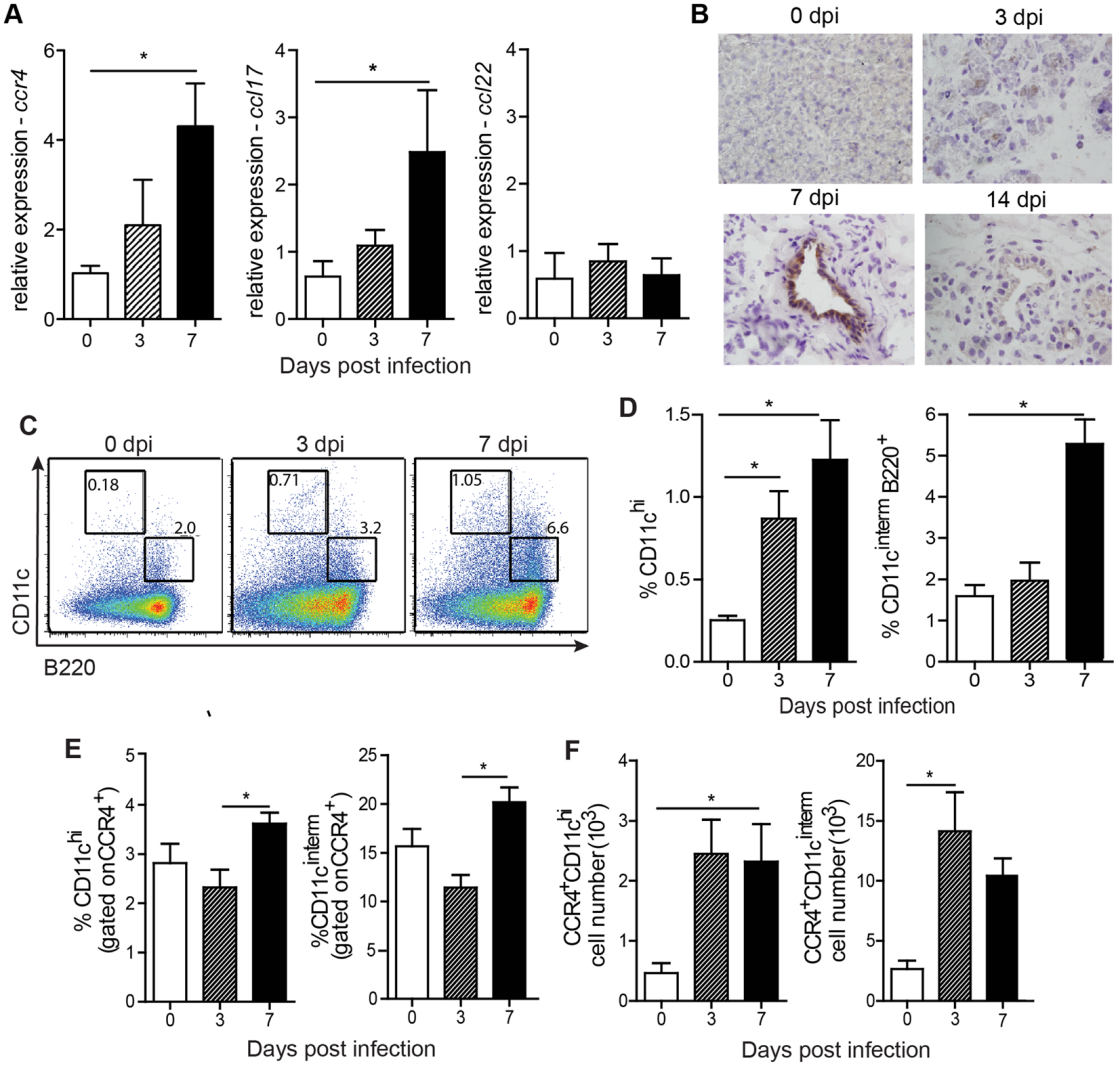
CVB5 induces the accumulation of CCR4^+^ DCs in the pLN. C57BL/6 mice were infected with CVB5, and the pancreas was harvested on days 0, 3, 7 and 14 (**A**,**B**). (**A**) qPCR for Ccr4, Ccl17 and Ccl22 expression. N = 5–6 mice per group. (**B**) Staining of CCL17 by immunohistochemistry. pLN were harvested on days 0, 3 and 7, and dendritic cells were analyzed by flow cytometry N = 6 mice per group. (**C**–**F**) Representative dot plot (**C**) and percentage (**D**) of CD11c^hi^ and CD11c^interm^B220^+^ dendritic cell populations. (**E**) Percentage of the same cell populations gated on CCR4^+^ cells. (**F**) Absolute number of CCR4^+^ dendritic cell populations. (**C**–**F**) N = 4 mice per group. All data are shown as the means + SEM from at least 3 independent experiments, and * indicates p < 0.05.

To investigate whether CCR4 and its ligands play a role during CVB infection, we first evaluated their expression in the pancreas, where virus presents a strong tropism^21^. Thus, C57BL/6 mice were infected i.p. with 10^6^ TCID_50_ of CVB5, and the pancreas was harvested on days 0, 3 and 7 post-infection for qPCR analysis. We found that the expression of *Ccr4* increased two-fold on day 3 and five-fold on day 7 post-infection. Consistently, expression of the CCR4 ligand, *Ccl17*, followed a similar up-regulation upon CVB5 infection. *Ccl22*, another CCR4 ligand, remained unaffected in the pancreas of infected mice for at least 7 days (Fig. 1A). The production of CCL17 protein was confirmed by immunohistochemistry, which showed intense staining of this chemokine in pancreatic duct cells 7 days after infection. After 14 days, CCL17 was no longer found in infected tissues (Fig. 1B), showing an acute production of CCL17 and CCR4^+^ cell accumulation.

DCs are important cells for IFN-I production and are key antigen presenting cells responsible for activation and polarization of adaptive immunity. Since these characteristics are crucial for viral immunity, and DCs were previously found to express CCR4^18^, we evaluated the migration of these cells to PLN throughout the infection. We found an accumulation of total CD11c^+^ cells in singlet population and both conventional (cDC) and plasmacytoid (pDC) subpopulations of DCs in the PLN after infection. The frequency of conventional DCs, represented by CD11c^hi^B220^−^ cells, was increased six-fold during infection in the PLN, for which migration started early, at day 3. The frequency of plasmacytoid DCs, represented by CD11c^interm^B220^+^ cells, increased approximately 3-fold; however, it started later, on day 7 post-infection (Fig. 1C,D). The percentage of both populations of DCs among CCR4^+^ cells was also increased in the PLN (Fig. 1E), and their absolute numbers were significantly increased early during CVB5 infection (Fig. 1F).

**Figure 2:**
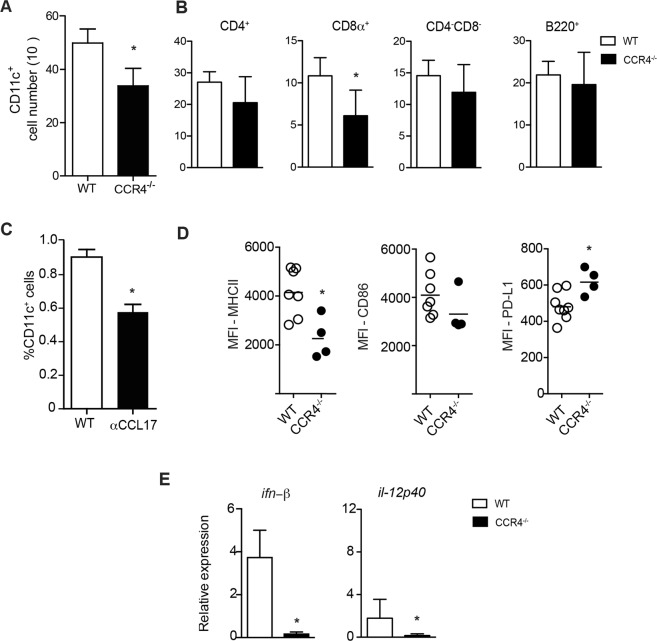
CCR4 induces DC migration and activation upon CVB5 infection. C57BL/6 and CCR4^−/−^ mice were infected with CVB5, and DCs were analyzed in the PLN at day 7 by flow cytometry (**A**,**B**,**D**). (**A**) Total number of CD11c^+^ cells and (**B**) CD4^+^, CD8α^+^, CD4^−^CD8^−^, and B220^+^ DC subpopulations. N = 6 mice per group. (**C**) C57BL/6 mice were treated on days −1, 1, 3 and 5 with anti-CCL17 antibody, and the percentage of total CD11c^+^ cells in singlet population was analyzed in the PLN 7 days post-infection. N = 4–5 mice per group. (**D**) Mean fluorescence intensity (MFI) of CD86, MHC-II and PD-L1 was evaluated among CD11c^+^ cells. N = 4–7 mice per group. (**E**) Expression of IFN-β and IL-12p40 was evaluated in pancreas homogenate by qPCR and related to *gapdh* as housekeeping gene. N = 4–6 mice per group. The data are shown as the means + SEM from at least 3 independent experiments, and * indicates p < 0.05.

To understand whether CCR4 is responsible for DC migration upon CVB5 infection, we infected CCR4^−/−^ mice and evaluated DC accumulation in the PLN on day 7. In the absence of CCR4, total CD11c^+^ cells accumulated less in the PLN (Fig. 2A). When we analyzed different subpopulations of DCs based on the expression of CD4, CD8α, and B220, we found a specific decrease in the number of CD8α^+^ DCs in CCR4^−/−^ mice (Fig. 2B). Treatment of C57BL/6 mice with anti-CCL17 antibody induced a similar decrease in CD11c^+^ cells in the PLN (Fig. 2C), suggesting that CVB5 induces DC migration in a CCL17 and CCR4-dependent manner.

DCs rely on their ability to migrate to infected tissue-draining lymph nodes and on their activation status to exert their functions on adaptive immune stimulation and anti-viral responses. We found that CCR4 is also important for DC activation, as DCs from CCR4^−/−^ mice presented lower expression of MHC-II and higher expression of the inhibitory molecule PD-L1 (Fig. 2D). In addition, the expression of IFN-β and IL-12p40 presented a profound reduction in CCR4^−/−^ mice after 7 days of infection (Fig. 2E). Taken together, the data show that CCL17 is produced in the pancreas upon CVB5 infection and that CCR4^+^CD11c^+^ cells migrate to infected tissue-draining lymph nodes. CCR4 is important for DC migration as well as for their activation and IFN-I and IL-12 production.

### CCR4^+^ DCs induce Th1 responses and provide resistance to CVB5-induced pancreatitis

**Figure 3:**
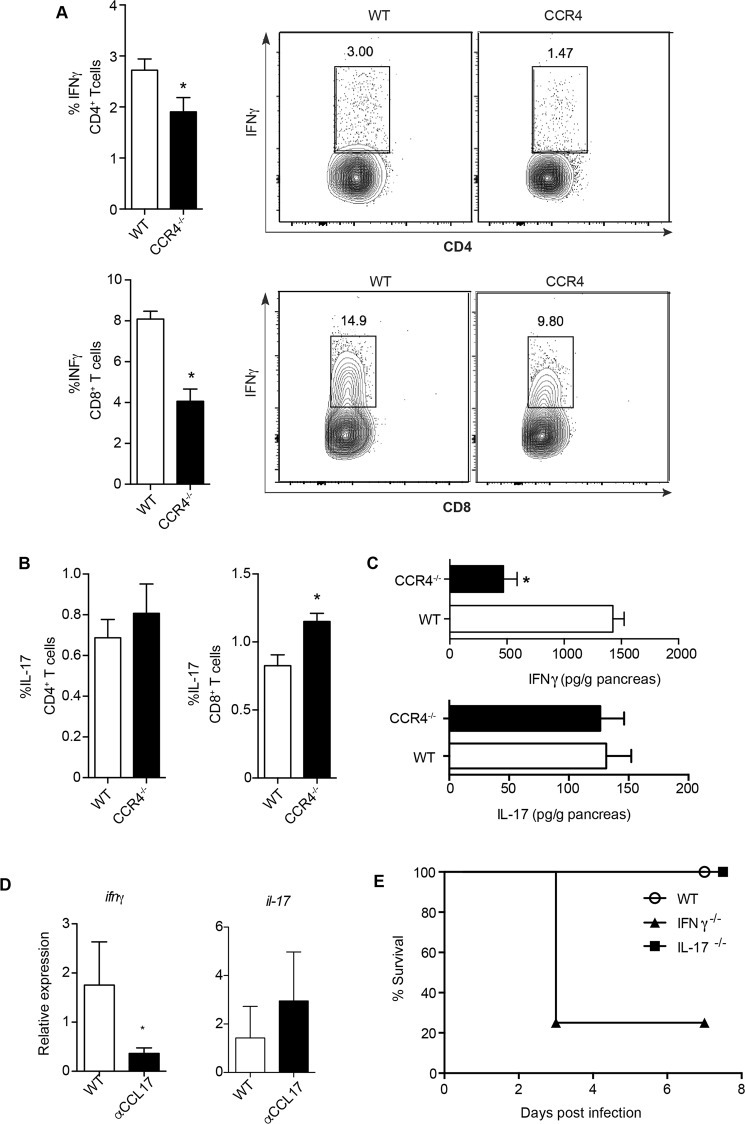
CCR4 induces Th1 polarization upon CVB5 infection. C57BL/6 and CCR4^−/−^ mice were infected with CVB5 (**A**–**D**). (**A**,**B**) The production of IFN-γ and IL-17 by CD4^+^ and CD8^+^ T cells was evaluated at day 7 post infection by flow cytometry after *ex vivo* stimulation with PMA and ionomycin in the presence of brefeldin-A. (**C**) IFN-γ and IL-17 were measured in the pancreas homogenate by ELISA at day 7. (**D**) C57BL/6 mice were treated on days −1, 1, 3 and 5 with anti-CCL17 antibody, and the expression of *ifng* and *il17* was evaluated in the pancreas at 7 days post-infection. (**E**) Survival of C57BL/6 (WT), IFN-γ^−/−^ and IL-17^−/−^ mice after CVB5 infection. N = 5–10 mice per group. The data are shown as the means + SEM from at least 3 independent experiments, and * indicates p < 0.05.

CD8α^+^ DCs are known to promote cross-presentation of antigen to CD8^+^ T cells as well as to polarize CD4^+^ T cells towards a Th1 profile^22,23^. The downregulation of IL-12p40 expression and the reduced accumulation of CD8α^+^ DCs in the PLN of CCR4^−/−^ mice after CVB5 infection prompted us to question whether Th1 responses are affected by CCR4. We found that the production of IFN-γ was reduced by both CD4^+^ and CD8^+^ T cells in the PLN of CCR4^−/−^ mice (Fig. 3A). In contrast, the production of IL-17 was increased by CD8^+^ T cells. The production of this cytokine by CD4^+^ T cells remained unaltered (Fig. 3B). In addition, the presence of IFN-γ was strongly reduced in pancreas homogenates of CCR4^−/−^ mice; however, IL-17 secretion was unchanged (Fig. 3C). Accordingly, the treatment of mice with anti-CCL17 antibody reduced the expression of *ifng* in the pancreas tissue after CVB5 infection, with no change on the expression of *Il17* (Fig. 3D). Whereas IL-17 seemed to be irrelevant to resistance to CVB5 infection, IFN-γ proved to be crucial, as 80% of IFN-γ^−/−^ mice succumbed as early as 3 days post infection (Fig. 3E). Together, the data show a role for CCR4 in promoting Th1 responses to CVB5, which are essential to confer resistance and survival.

**Figure 4:**
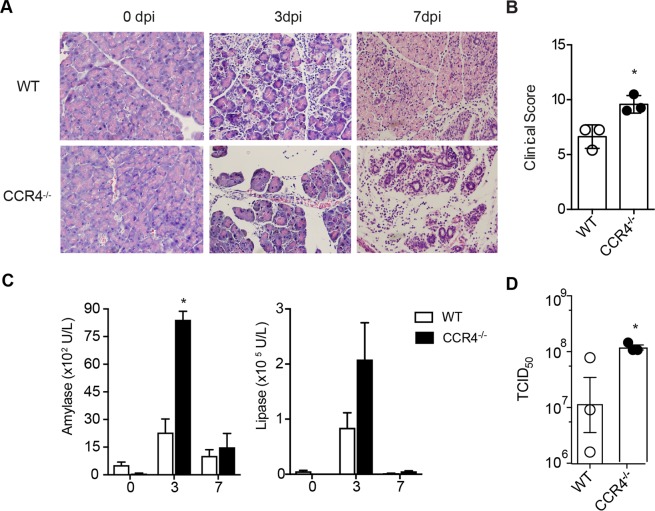
CCR4 provides resistance to CVB5-induced pancreatitis. (**A**) Pancreas sections from C57BL/6 (WT) and CCR4^−/−^ mice stained with H&E on days 0, 3 and 7 post-CVB5 infection (dpi). (**B**) The clinical score was determined by the level of inflammation, edema and necrosis at day 7 post infection. (**C**) Amylase and lipase were measured in the serum. (**D**) Viral titers were measured in pancreas homogenates by TCID_50_ on day 3. N = 5–7 mice per group. The data are shown as the means + SEM from at least 3 independent experiments, and * indicates p < 0.05.

To investigate whether CCR4 is important for the resistance of mice to CVB5 infection, we analyzed virus-induced pancreatitis in CCR4^−/−^ mice. Although CCR4^−/−^ mice survived the infection (data not shown), their pancreata were more injured than WT mice, presenting significantly more inflammatory infiltrates, edema and necrosis on days 3 and 7 post-infection (Fig. 4A,B). In addition, the levels of amylase and lipase in the serum of infected CCR4^−/−^ mice, as a measure of pancreatic damage, were dramatically higher than those in WT mice (Fig. 4C). This correlated with higher virus titers in CCR4^−/−^ mice at the same time point (Fig. 4D), showing that CCR4 plays a significant role in conferring resistance to CVB5-induced pancreatitis.

**Figure 5:**
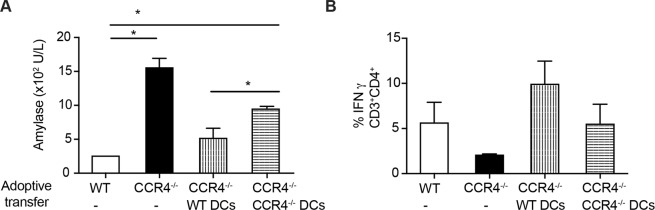
CCR4^+^ DCs induce Th1 responses and provide resistance to CVB5-induced pancreatitis. WT or CCR4^−/−^ BMDCs (10^6^) were adoptively transferred into CCR4^−/−^ mice on day 1, and the mice were then infected with CVB5. Amylase was measured in the serum on day 3 post-infection (**A**), and the production of IFN-γ by CD4^+^ T cells was determined in PLN on day 7. (**B**) N = 4–5 mice per group. The data are shown as the means + SEM from 2 independent experiments, and * indicates p < 0.05.

To identify the CCR4-expressing cells that induce Th1 immune responses and confer resistance to CVB5 infection, we differentiated bone marrow-derived DCs in culture (BMDCs) from WT and CCR4^−/−^ mice and adoptively transferred them into CCR4^−/−^ mice 1 day after CVB5 infection to allow them to migrate to the infected tissue-draining lymph nodes. We then analyzed the pancreatitis and Th1 cytokine production. As previously seen, CCR4^−/−^ mice presented elevated serum amylase, which correlated with reduced IFN-γ production compared with WT mice. When WT DCs were transferred into CCR4^−/−^ mice, the amylase level was reduced to a level similar to that of WT (Fig. 5A), and the IFN-γ production by CD4^+^ T cells was up-regulated similarly as in WT mice. In contrast, transfer of CCR4^−/−^ DCs did not change the IFN-γ production (Fig. 5B). Although CCR4^−/−^ DCs were able to reduce the amylase level in serum, they were not as efficient as CCR4^+^ DCs (Fig. 5A). Collectively, the data show that CCR4^+^ DCs are responsible for the polarization of T cells towards a Th1 profile and confer resistance to CVB5-induced pancreatitis.

### CCR4^+^ tregs, but not CCR4^−^ tregs, regulate T cell activation upon CVB5 infection

**Figure 6:**
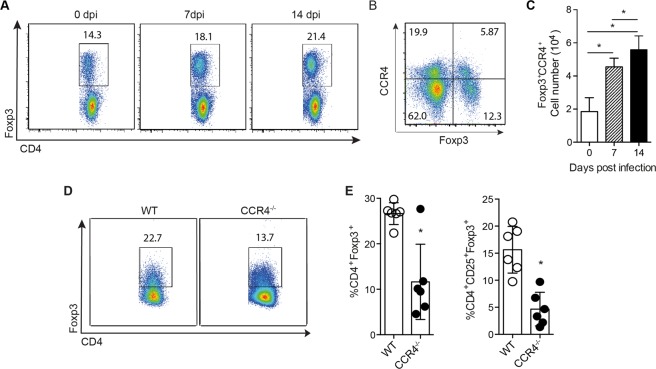
Tregs are induced upon CVB5 infection through CCR4. Foxp3-GFP mice were infected with CVB5, and Tregs were analyzed in the PLN on days 0, 7 and 14 post-infection by flow cytometry (**A**,**C**). (**A**) Representative dot plot of CD3^+^CD4^+^Foxp3^+^ cells. (**B**) Representative dot plot of Foxp3^+^CCR4^+^ cells at day 7 post infection. (**C**) Absolute number of CCR4^+^Foxp3^+^CD3^+^CD4^+^ cells (**D**,**E**) Tregs from C57BL/6 (WT) and CCR4^−/−^ mice infected with CVB5. (**D**) Representative dot plot of Foxp3^+^ cells in CD3^+^CD4^+^ gate. (**E**) Percentage of Foxp3^+^ and CD25^+^Foxp3^+^ cells within CD3^+^CD4^+^ cells. N = 3–6 mice per group. The data are shown as the means + SEM from at least 3 independent experiments, and * indicates p < 0.05.

We and others have previously shown that pancreatitis induced by CVB5 infection is due to both viral replication and exacerbated immune activation. T regulatory cells (Treg) are key in controlling the activation of immune responses in several scenarios, including during CVB5 infection, where they inhibit pancreatic damage caused by inflammation^14–16^. In addition, Tregs were shown to express CCR4 in response to tumors and inflammation^19,24,25^. To verify that CCR4 expression is induced in Treg throughout CVB5 infection, we analyzed Foxp3^+^ cells in the PLNs on days 0, 7 and 14 post-infection. As expected, the frequency of CD3^+^CD4^+^Foxp3^+^ T cells increased in the PLN after 7 and 14 days post-infection (Fig. 6A). In addition, the number of Foxp3^+^ cells expressing CCR4^+^ was also increased at day 7 post infection (Fig. 6B). When CCR4^−/−^ mice were infected with the virus, we found a reduced percentage of Tregs in the PLN compared with WT by analyzing both Foxp3^+^ and CD25^+^Foxp3^+^ cells within CD3^+^CD4^+^ population (Fig. 6C,D). These data show that Tregs are induced or migrate to the PLN upon CVB5 in a CCR4-dependent manner.

**Figure 7:**
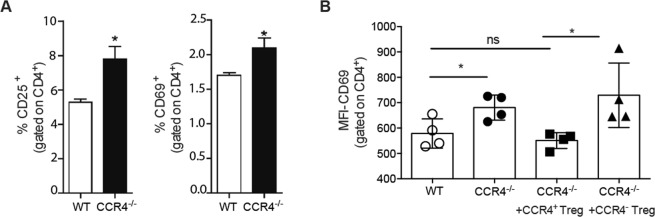
Tregs expressing CCR4 inhibit T cell activation upon CVB5 infection. (**A**) The percentage of CD25^+^ and CD69^+^ on CD3^+^CD4^+^ cells in the PLN 7 days post-CVB5 infection. (**B**) Foxp3^+^CCR4^+^ (CCR4^+^ Treg) and Foxp3^+^CCR4^−^ (CCR4^−^ Treg) cells were sorted from the PLN of Foxp3-GFP mice infected with CVB5. Cells were adoptively transferred into CCR4^−/−^ mice on day 4 post-CVB5 infection. At day 7, PLNs were harvested, and the expression of CD69 was analyzed on CD3^+^CD4^+^ cells by mean fluorescence intensity (MFI). N = 4–5 mice per group.

We next questioned whether the failure to induce Treg in the absence of CCR4 would culminate in defective immune regulation. Indeed, we found that CCR4^−/−^ mice presented an increased frequency of CD4^+^ T cells activation, as seen by CD25 and CD69 expression, in the PLN after CVB5 infection (Fig. 7A), whereas there was no difference in CD4^+^ T cells activation of WT and CCR4^−/−^ naive mice (data not shown). To confirm the role of Tregs and CCR4 on T cell regulation, we infected Foxp3-GFP mice with CVB5 and sorted CCR4^+^Foxp3^+^ and CCR4^−^Foxp3^+^ Treg populations. The cells were adoptively transferred into CCR4^−/−^ mice after 4 days of CVB5 infection, and T lymphocyte activation was analyzed on day 7. Day 4 was chosen to perform the Treg transfer based on the virus replication, which peaks at day 3; therefore, the immune system might be able to respond to and contain the virus before immunity is initiated. As expected, CCR4^−/−^ mice had higher CD69 expression by CD4^+^ T cells compared with WT mice. When CCR4^+^ Tregs were transferred into CCR4^−/−^ mice, CD69 expression was significantly reduced to the same level as WT mice. In contrast, CCR4^−^ Tregs were not able to change CD69 expression by CD4^+^ T cells (Fig. 7B), showing that CCR4^+^ Tregs are responsible for regulating CD4^+^ T lymphocyte activation.

Altogether, the present work shows a CCR4^+^ cells migration upon CVB5 infection. The accumulation of CD11c^+^ cells in pancreatic lymph nodes (PLN), especially the Th1-inducing CD8α^+^ DCs, and their activation and production of IFN-β and IL-12 were CCR4-dependent. Consequently, CCR4^−/−^ mice had reduced IFN-γ-producing T cells in the pancreas and lymph nodes and were more susceptible to CVB5-induced pancreatitis. Tregs were also induced or recruited to the PLN after CVB5 infection, and CCR4^+^ Tregs, but not CCR4^−^ Tregs, were responsible for regulating T lymphocyte activation, which correlated with increased resistance to pancreatitis.

## Discussion

Cardiac and pancreatic injury induced by CVB infection is caused by both direct virus replication and immune-mediated damage, which together induce cell lysis, edema and tissue fibrosis^3^. Thus, the coordination and regulation of immune responses are key factors for elimination of the virus with minimal harm to the host. For this, migration of the appropriate cells to the site of infection is extremely important for the resolution of the disease, which is mainly coordinated by chemokines^26,27^.

In the present work, we have shown that intraperitoneal infection of mice with CVB5 induced pancreatic duct cells to produce the chemokine CCL17, whereas the expression of its receptor, CCR4, was increased in the pancreas, and CCR4^+^ cells accumulated in the PLN. Although some studies have screened the expression of different chemokines during CVB infection^28^, our data are the first to indicate the involvement of CCL17 in the recruitment of cells expressing CCR4 to the pancreas and PLN during infection. Unlike a previous study that observed the production of CCL22 in the myocardium after CVB3 infection^29^, we did not find CCL22 expression in the pancreas after acute CVB5 infection, indicating differences in the recruitment of cells during infection with different strains of Coxsackievirus to different tissues.

Among the cells that express CCR4 are DCs, which are immunological sentinels, fundamental for the homeostasis of T cell-dependent responses^30^. Our data show that, upon CVB5 infection, there is an increase in both the CD11c^hi^ cell population, characterized by conventional DCs, and the CD11c^interm^B220^+^ cell population, characterized by plasmacytoid DCs, in the PLN. In addition, the migration of these two populations of DCs expressing CCR4 was intensively increased 3 and 7 days post-infection. Although CCL17 is strongly expressed only at 7 days post-infection and we cannot exclude the possibility that other mediators cooperate in DC migration, the data suggest the involvement of CCR4 in the recruitment of these cells. Since we used the intraperitoneal route of infection, which skips the gastric phase of the virus infected by the natural oral route, the kinetic of migration of the cells may be delayed in the natural infection.

This hypothesis was confirmed upon CVB5 infection of CCR4^−/−^ mice. In mice that lack CCR4, the frequency and number of dendritic cells in the PLN was significantly lower than those in WT mice. Treatment of WT mice with anti-CCL17 antibody had a similar effect, suggesting the role of the CCR4-CCL17 axis in DC recruitment to the PLN. Furthermore, we found that CD8α^+^ DCs are the subpopulation whose migration is CCR4-dependent. CD8α^+^ DCs are able to cross-present antigens via MHC class I to CD8^+^ T cells as well as skew CD4^+^ T cells towards a Th1 profile by producing IL-12^31^. It has been shown that the expansion of CD8α^+^ DCs confers protection during myocarditis caused by CVB3^32^. Thus, our data point to CCR4 as important molecule involved in the recruitment of cells responsible for the resolution of infection.

IFN-I are important cytokines for the elimination of viral infections^33,34^, and CCR4^−/−^ mice had reduced expression of IFN-β, which correlated with a higher viral load; thus, IFN-β secretion may be partially responsible for viral clearance early in infection when the adaptive immune response is not yet shaped. In addition, it has been demonstrated that IFN-I produced at the beginning of infection is responsible for the effective response of NK and CD8^+^ T cells against diverse acute viral infections^35,36^. CD8α^+^ DCs together with plasmacytoid DCs are capable of IFN-I production, although in smaller concentrations; thus, the source of IFN-I that is induced by CCR4 still remains to be determined.

In addition to inducing cell recruitment, chemokines are also capable of activating cells^37^. Thus, we investigated the expression of activating and inhibitory markers on DCs, which reflect the ability to present antigen and stimulate T lymphocytes^38^. Our results show that, during both *in vitro* (data not shown) and *in vivo* infections, CCR4^−/−^ DCs exhibited decreased expression of MHC class II and the co-stimulatory molecule CD86 as well as increased expression of the inhibitory molecule PD-L1, indicating that CCR4 is important for the activation of DCs after CVB5 infection.

In line with these data, in the absence of CCR4, both CD4^+^ and CD8^+^ T cells produced less IFN-γ after infection by CVB5, an important cytokine in the immune response against viruses, and CD8^+^ T cells produced more IL-17. IL-17 production during CVB3 infection has been shown to worsen tissue injury, possibly due to ineffective viral clearance^39–41^, and neutralization of IL-17 with antibodies was able to reduce virus-induced myocarditis^42^. Consequently, CVB5 infection of CCR4^−/−^ mice aggravated the pancreatitis, as the pancreas presented more intense inflammatory infiltrates, edema and cell necrosis. Thus, our data show that CCR4 is important for the induction of an adequate adaptive immune response during CVB5 infection by skewing T cells toward a protective Th1 pattern that is capable of controlling viral replication with minor pancreatic damage. By adoptive transfer of cells, we demonstrated that CCR4^+^ DCs are, at least in part, responsible for inducing CD4^+^ and CD8^+^ T cells-mediated IFN-γ production and for controlling the pancreatic injury, although CCR4^−^ DCs may also play a role in the impairment of pancreatitis development.

We further showed that the induction or migration of Tregs is also affected by CCR4, as CCR4^−/−^ mice had decreased frequency and absolute numbers of Tregs in the PLN after CVB5 infection compared to WT mice. Our data showing CCR4 as important for the recruitment of Tregs to the target tissue corroborate those obtained by previous works that correlated the expression of CCR4 to Treg migration to the lung and skin in experimental models of airway allergic inflammation and fungal infection, respectively^25,43,44^.

Previous work has shown that the higher resistance conferred by female mice to CVB3 infection is closely related to the presence of Tregs in the myocardium^11^. In addition, we also previously showed that the presence of Tregs in the PLN is involved in the protection against CVB5 injury, as the inhibition of Treg functions by treatment with anti-GITR antibody increased the inflammatory infiltrate and necrosis in the pancreas^16^. The present data show that the decrement of Treg numbers in CCR4^−/−^ mice was directly correlated with higher percentages of activated T cells, as seen by CD25 and CD69 expression. Moreover, adoptive transfer of CCR4^+^ Tregs, but not CCR4^−^ Tregs, into CCR4^−/−^ mice completely restored the level of activation of T cells seen in WT mice. It remains uncertain, however, whether CCR4^−^ Tregs are ineffective because they are unable to migrate to the PLN, which would promote CCR4 as the major receptor responsible for Treg migration in the present model, or by CCR4 also playing a role in promoting one of the diverse mechanisms of suppression exerted by Tregs, as it has been shown that CCR4 also influences the suppressive effects of Tregs^45,46^. In addition, since CCR4^−/−^ mice have lower DC activation, the inefficient activation of CVB-specific T lymphocytes in these mice might also play a role on the reduction of Treg migration to PLN.

In conclusion, the data show the essential role of the CCR4 pathway in the recruitment of cellular subpopulations important for viral restraint and simultaneously for control of the inflammatory response induced by CVB infection. We implicate DCs and Tregs as major CCR4-expressing cells in this process, although we cannot exclude the contribution of CCR4 in the migration of other cells types to the lesion. The murine model of CVB5-induced pancreatitis shares many characteristics of the disease in humans. Chronic pancreatitis is also a risk factor for pancreatic cancer, one of the most debilitating untreated diseases to date. Our data reveal a previously unknown mechanism by which cells important for the resolution of infection are able to migrate to the site and perform their functions, and our data point to CCL17 and CCR4 as potential targets for the treatment of viral pancreatitis.

## Materials and Methods

### Mice and antibody treatment

C57BL/6, Foxp3-GFP, CCR4^−/−^, IFN-γ^−/−^ and IL-17^−/−^ mice on the C57BL/6 background were purchased from the Jackson Laboratory (Bar Harbor, ME, USA) and were maintained in the mouse facility of School of Medicine of Ribeirão Preto, University of Sao Paulo in temperature-controlled rooms with water and food provided *ad libitum*. In some experiments, the mice were treated i.p with 10 μg/day of α-CCL17 antibody (R&D Systems, Minneapolis, MN, USA) on days −1, 1, 3 and 5 days post-infection (dpi). The animal protocol used was approved by the Ethical Commission of Ethics in Animal Research of the University of São Paulo, Ribeirão Preto School of Medicine (Protocol No. 198/2009). This commission is part of the National Brazilian College of Animal Experimentation. We confirm that all methods were performed in accordance with the relevant guidelines and regulations.

### Virus titration and infection

The original stock of CVB5, kindly provided by Prof. Eurico Arruda Neto, was propagated in a Vero cell monolayer in MEM (Life Technologies, Carlsbad, CA, USA) supplemented with 2% FBS and antibiotics at 37 °C. The virus was titrated by limiting dilutions and inoculated in quadruplicate to determine the 50% cytopathic effect of the cellular monolayer (TCID_50_). The C57BL/6, CCR4^−/−^, IFN-γ^−/−^ and IL-17^−/−^ mice were injected i.p. with either 10^6^ TCID_50_ CVB5 in 100 μl PBS or Vero cell supernatants alone as a negative control. The animals were sacrificed at 0, 3, 7 or 14 dpi. We have chosen the intraperitoneal route since we infected adult C57BL/6 mice, which are more resistant to infection, thus the virus was delivery directly to the target tissue. This route is broadly used and it is shown to mimic the acute pancreatic infection caused by the virus.

### Histology and scores

The histology and clinical scores were performed as previously described^16^. Briefly, the pancreata from the mice were fixed in paraffin and sliced into 5 µm thick sections, which were assembled on glass slides and stained with H&E. The clinical score classified the inflammatory infiltrated cell area and the edema/necrosis of the infected tissue in a blinded manner.

### Immunohistochemistry

The pancreas from C57BL6 mice was harvested 0, 3 and 7 days post-infection and was frozen in *Tissuetek O*.*T*.*C*. The tissue sections were first fixed in P.A. acetone for 20 min at −20 °C and then incubated with PBS/1% BSA to block non-specific binding. The following incubation included the goat α-CCL17 antibody diluted 1:100 (Santa Cruz Biotechnology, Dallas, TX, USA) in PBS 0.01% saponin overnight. The secondary antibody (rabbit α-goat conjugated to biotin diluted 1:400) was added in PBS and incubated for 2 hours. The sections were then incubated with avidin-biotin-HRP complex (Vector Laboratories, Burlingame, CA, USA) for 30 minutes, and the reaction was revealed with DAB chromogen solution. The tissue sections were washed 3 times in PBS between each incubation step. The tissue was counterstained with H&E, mounted in mounting medium and covered with a coverslip.

### Cell transfer

Bone marrow-derived dendritic cells (BMDC) were differentiated in culture for 7 days in RPMI 1640 containing 10% FBS, 2 mM L-glutamine, 100 U/ml penicillin, 100 mg/ml streptomycin and 20 ng/ml GM-CSF. On day 4, the medium with GM-CSF was replenished. The BMDCs (10^6^ cells) were transferred i.v. to the recipient mice on day 1. The purity of BMDCs was routinely above of 90%.

For the experiments with Treg transfers, the Foxp3-GFP mice were infected with CVB5. On day 7, the spleen and PLN cells were harvested and stained with anti-CCR4 antibody conjugated to APC. The CCR4^+^GFP^+^ and CCR4^−^GFP^+^ Treg populations were sorted using a FACSAria (BD Bioscience, San Diego, CA, USA) and were transferred i.v into the CVB5-infected CCR4^−/−^ mice on day 4 post-infection. Analysis of CD69 expression levels in T cells was performed on PLN cells on day 7.

### Detection of cytokines and serum enzymes

The pancreas homogenates were examined for IFN-γ and IL-17 by ELISA according to the manufacturer’s instructions (R&D Systems, Minneapolis, MN, USA). Amylase and lipase were determined in the serum of mice 3 days post-infection by a standard colorimetric assay according to the manufacturer’s instructions (Labtest).

### RNA extraction and qPCR

RNA from the pancreas was extracted using the SV Total RNA Isolation System (Promega, Madison, WI, USA) according to the manufacturer’s instructions and reverse-transcribed using the High Capacity cDNA Reverse Transcription Kit (Applied Biosystems, Foster City, CA, USA). The expression of *Ccr4*, *Ccl17*, *Ccl22*, *Ifnβ* and *Ifnγ* was analyzed by qPCR using SYBR Green PCR Master Mix (Applied Biosystems, Foster City, CA, USA), and *Gapdh* was used as an internal control for normalization. The data were analyzed using the ΔCT method (cycle threshold of test – cycle threshold of endogen control). The following primers were used: *Ccr4* forward: AAGGCAGCTCAACTGTTCTCATT, reverse: TGGTGTCTGTGACCTCTGTGG; *Ccl17* forward: GAAGTCCCTGTTCCCTTTTTT, reverse: TGTGTTCGCCTGTAGTGCATA; *Ccl22* forward: ATGGTGCCAATGTGGAAGA, reverse: TAAACGTGATGGCAGAGGGT; *Ifnb* forward: CATCAACTATAAGCAGCTCCA, reverse: TTCAAGTGGAGAGCAGTTGAC; *Gapdh* forward: TGCAGTGGCAAAGTGGAGAT, reverse: CGTGAGTGGAGTCATACTGGAA.

### Flow cytometry

Surface staining was performed on PLN cells incubated with 10% rabbit serum to block non-specific binding for 20 min and then incubated with anti-CD3, CD4, CD8, CD11c, B220, MHC-II, CD86, PD-L1, CD25, CD69, and CCR4 conjugated to the following fluorochromes: PE, PE-Cy7, FITC, APC, APC-Cy7 or PerCP. For the Foxp3 staining, the cells were fixed and permeabilized using the BD Pharmingen mouse Foxp3 buffer set (BD Bioscience, San Diego, CA, USA) and then incubated with anti-Foxp3 antibody conjugated to PE for 30 min. For the intracellular cytokine staining, PLN cells were incubated with PMA (500 μg/ml), ionomycin (50 μg/ml), and GolgiStop (BD Biosciences, San Diego, CA, USA) for 6 h at 37 °C. Extracellular staining was performed, followed by fixation and permeabilization using the Cytofix/Cytoperm kit (BD Bioscience, San Diego, CA, USA). Anti-IFN-γ and IL-17 antibodies conjugated to PE or APC were added to the cell suspension and incubated for 30 min. The cells were acquired on a flow cytometer (FACS CantoII; BD Biosciences). Data analysis was performed using FlowJo software (Tree Star, Ashland, OR).

### Statistical analysis

The data were analyzed using Student’s *t* test for comparison of two experimental groups and variance analysis (ANOVA) followed by Bonferroni’s test for comparison of more than two groups. A *p* value < 0.05 was considered statistically significant and is represented by * in the figures. All the error bars indicate standard error (SEM).
